# Efficacy and safety of interleukin-17A inhibitors in patients with ankylosing spondylitis: a systematic review and meta-analysis of randomized controlled trials

**DOI:** 10.1007/s10067-020-05545-y

**Published:** 2021-01-12

**Authors:** Peng Wang, Shuo Zhang, Binwu Hu, Weijian Liu, Xiao Lv, Songfeng Chen, Zengwu Shao

**Affiliations:** 1 Department of Orthopaedics, Union Hospital, Tongji Medical College, Huazhong University of Science and Technology, Wuhan, 430022 Hubei Province China; 2 Department of Orthopaedics, The First Affiliated Hospital of Zhengzhou University, Zhengzhou, 450052 China

**Keywords:** Ankylosing spondylitis, IL-17A inhibitors, Meta-analysis, Randomized controlled trial

## Abstract

To assess the efficacy and safety of interleukin (IL)-17A inhibitors in patients with ankylosing spondylitis (AS). PubMed, EMBASE, and Web of Science were searched up to 5 February 2020 for randomized controlled trials (RCTs) that assessed the efficacy and safety of IL-17A inhibitors in patients with AS. We used a meta-analytic approach to perform a random effects analysis or fixed effects analysis according to heterogeneity. Subgroup analyses between studies included medication, time to primary endpoint, and data source. Odds ratios (ORs) or mean differences (MDs) were used to assess the efficacy and safety of IL-17A inhibitors in AS. A total of ten RCTs with 2613 patients were eligible for inclusion in the analysis (six for secukinumab, two for ixekizumab, one for netakimab, and one for bimekizumab). Compared to placebo, IL-17A inhibitors improved ASAS20 response rate (OR = 2.58; *p* < 0.01) and ASAS40 response rate (OR = 2.80; *p* < 0.01), and significantly increased the risk of AEs (OR = 1.23; *p* = 0.03) and nasopharyngitis (OR = 1.72; *p* < 0.01), but not SAEs (OR = 0.87; *p* = 0.60). IL-17A inhibitors demonstrated better efficacy in patients with AS in several evaluation indicators. However, the safety of IL-17A inhibitors remains to be further studied in studies with larger sample size and longer follow-up times.

## Introduction

Ankylosing spondylitis (AS), also termed radiographic spondyloarthritis, is a primary subtype of axial spondyloarthritis. AS is a chronic inflammatory rheumatic disease, which is featured with axial inflammation, and imaging-visible structural damages in the spine and sacroiliac joints [1–3]. With an incidence of 0.02–0.35% [4], AS not only decreases the work productivity, and life quality [5], but also results in heavy burdens of public health care all over the world [6]. The molecular and pathogenesis mechanisms that underlie AS still have many dark sides. Although the exact etiology and pathogenesis mechanisms are not clear, many studies have instructed that the occurrence of AS is closely related to the positive expression of human leukocyte antigen (HLA)-B27 [7, 8]. Immune system also promotes the development and progression of AS, which can be characterized by overexpression of inflammatory cytokines and abnormal activation of immune cells in AS patients.

During the past decades, researchers have kept exploring the therapies for AS. Physical therapy is proved to be efficacious for AS patients. However, its application is limited due to high expense and low accessibility [9]. For AS patients suffering from pain and stiffness, non-steroidal anti-inflammatory drugs (NSAIDs) are recommended as the first-line treatment [10]. Recently, biological disease-modifying antirheumatic drugs (bDMARDs) emerged as novel therapies for AS. For example, tumor necrosis factor (TNF) inhibitors are recommended to patients with AS who are in persistent disease activity period [10]. Nevertheless, NSAIDs and TNF inhibitors are not always effective and well-tolerated for all types of AS patients [10, 11]. Therefore, novel therapeutic alternatives for AS are highly required.

Mounting studies have demonstrated that interleukin (IL)-23/IL-17 axis was highly associated with immune dysfunction and activated autoimmune inflammation. Further studies demonstrated that IL-23/IL-17 axis was involved in the pathogenesis of multiple rheumatoid diseases, including spondyloarthritis, psoriasis, rheumatoid arthritis, and inflammatory bowel diseases [12–14]. IL-17, also named as cytotoxic T lymphocyte antigen 8 (CTLA8), was first cloned from activated T cells in 1993 [15]. IL-17 family includes six members from IL-17A to IL-17F, of which IL-17A and IL-17F are crucial proinflammatory cytokines [16]. Soon after the discovery of IL-17, the IL-17 receptor (IL-17R) family was identified consisting of five members ranging from IL-17RA to IL-17RE [17]. The IL-17 members may combine with the IL-17Rs [18], and then activate various inflammatory pathways, including the nuclear factor κB (NF-κB) pathway, the mitogen-activated protein kinases (MAPKs) pathway, and the CCAAT/enhancer-binding proteins (C/EBPs) pathway [19]. The activation of these signal transduction pathways leads to the overexpression of various proinflammatory cytokines, such as IL-6, IL-8, TNF-α, and IL-1β [18]. Notably, in the individuals with AS, serum level of IL-17 [20] and IL-23 [21] as well as the number of T helper (Th) 17 cells [22–25] in peripheral blood and facet joints [26] were increased compared with healthy control subjects.

Based on a growing body of researches, IL-17A was recognized as a novel therapeutic target for AS. Biological IL-17A inhibitors, which are crucial members of bDMARDs, could directly combine with IL-17A and inhibit its function [27]. IL-17A inhibitors are well established and known as efficient and safe for the treatment of psoriasis [28], rheumatoid arthritis [29, 30], and inflammatory bowel diseases [31]. Recently, increasing randomized controlled trials (RCTs) [32–41] have investigated the efficacy and safety of IL-17A inhibitors in AS, but the clinical value of IL-17A inhibitors in AS is still an area of controversy due to different drug types and dosages. Previous meta-analysis on biological agents for AS treatment only included one or two studies about IL-17A inhibitors [42, 43], after that there were few newly established studies. As such, an updated quantification of the efficacy and safety of IL-17A inhibitors was warranted.

Herein, considering that a number of studies have demonstrated the impact of IL-17A inhibitors in AS, the present systematic review and meta-analysis were conducted to comprehensively understand the efficacy and safety profile of IL-17A inhibitors in AS.

## Materials and methods

This meta-analysis was performed in accordance with the Cochrane Handbook for Systematic Review of Interventions [44].

### Literature retrieval

A comprehensive literature retrieval was carried out in PubMed, EMBASE, and Web of Science up to February 5, 2020, by two reviewers independently. The search was performed using the terms “ixekizumab OR taltz OR LY2439821 OR secukinumab OR cosentyx OR AIN 457 OR bimekizumab OR UCB4940 OR netakimab OR Anti IL-17 OR Interleukin 17 OR IL-17 inhibitor OR Interleukin 17 inhibitor” AND “ankylosing spondylitis OR spondyloarthritis.” In addition, reference lists of the relevant articles and reviews were examined carefully to identify additional eligible articles.

### Selection criteria

Articles were included in this analysis if they met all the following criteria: (1) the study was a RCT; (2) patients were diagnosed as AS according to the modified New York criteria; (3) treatment groups received IL-17A inhibitors and control groups received placebo in the RCTs; (4) the efficacy or safety outcomes were reported in the RCTs; (5) the language of the articles was English. Additionally, we excluded animal studies, observational studies, reviews, commentaries, letters, and RCTs that examined other interventions. For the publications with repetitive trial numbers according to the ClinicalTrials.gov identifier, only the most recent one was included. Two authors independently screened all studies by title or abstract and then by a full text evaluation; any discrepancy was solved by discussion.

### Data extraction and quality assessment

Data extraction was carried out independently by two authors using a predefined form. We extracted the following information from each included study as follows: first author, publication year, NCT number, region, time to primary endpoint, study duration, medication and dosage, number of cases, mean age, ratio of males, disease duration, HLA-B27-positive rate, baseline data of the Bath AS disease activity index (BASDAI).

Quality assessment was performed using the Cochrane Collaboration’s tool. We assessed risk of bias according to the following bias categories: random sequence generation, allocation concealment, blinding, incomplete outcome data, selective reporting, and other bias. Each segment was assessed as high risk, low risk, or unclear risk of bias.

### Efficacy and safety outcomes

Primary efficacy outcomes were as follows: 20% improvement according to the Assessment of SpondyloArthritis international Society (ASAS) criteria (ASAS20 response), and 40% improvement according to ASAS criteria (ASAS40 response). Other efficacy outcomes were 20% or more improvement in five of the six ASAS response domains (ASAS5/6 response), the change from baseline in total BASDAI, and a score of ≤ 2 units in each of the four core ASAS domains (ASAS partial remission). Safety outcomes included adverse events (AEs), serious adverse events (SAEs), nasopharyngitis, discontinuation due to any AEs (DDAAEs), infections, and serious infections.

### Statistical analysis

Review Manager 5.3 was used to perform the data analysis. Odds ratios (ORs) with corresponding 95% confidence intervals (CIs) were considered as the effect size for dichotomous variables, and mean differences (MDs) with 95% CIs were for continuous variables. We performed Cochran I-squared test to assess the heterogeneity among studies. It was supposed that no significant heterogeneity existed among studies if *I*^2^ < 50%, and thus a fixed-effects model was applied. Otherwise, a random-effects model was considered to be more appropriate if *I*^2^ > 50%. To explore the source of heterogeneity among studies and to test the robustness of the results, we further conducted subgroup analyses by the following factors: medication (secukinumab, ixekizumab, netakimab, or bimekizumab), time to primary endpoint (6 weeks, 12 weeks or 16 weeks), and data source (full-text article or conference abstract). If included studies held several treatment arms in terms of dosage, we combined relevant treatment groups into one treatment group, as the Cochrane Handbook for Systematic Review of Interventions recommended [44]. Sensitivity analysis was conducted by omitting any of the studies at a time to further evaluate the stability and credibility of the analytical results. Statistical significance was defined as *P* value < 0.05.

## Results

### Study selection and characteristics of included studies

**Fig. 1:**
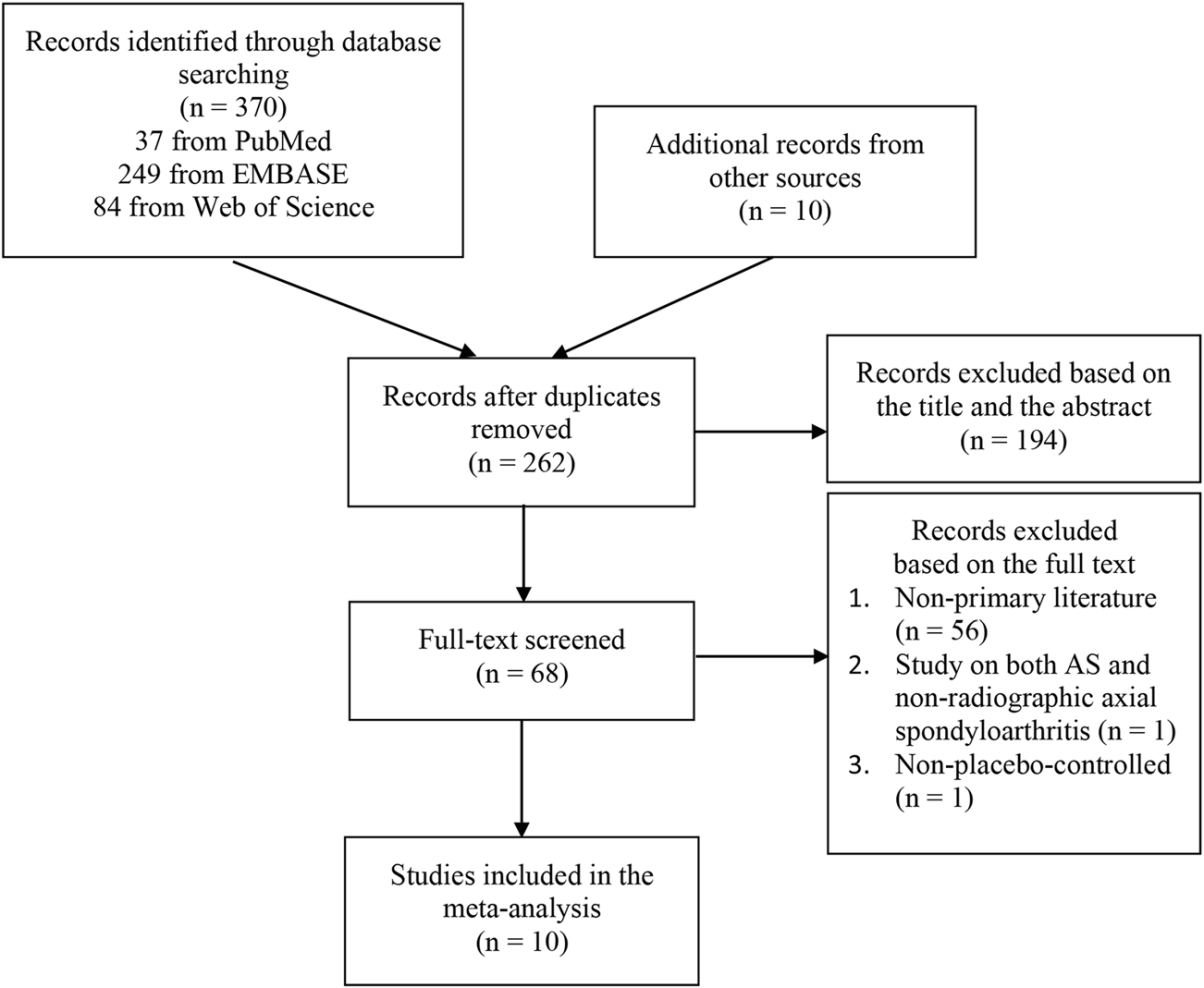
Flowchart of study selection process

The searching process was summarized in Fig. 1. According to the study searching strategy stated above, 370 records were identified through database searching and 10 records were obtained from the references of the identified articles. After removing 118 duplicated records, we screened the remaining 262 records and excluded 194 records according to the title and the abstract. Then, full texts of the remaining 68 articles were viewed carefully, and 58 of them were excluded. Finally, ten RCTs with 2613 patients were included in our meta-analysis in total, of which seven were published in articles and three were reported in conference abstracts.

**Fig. 2:**
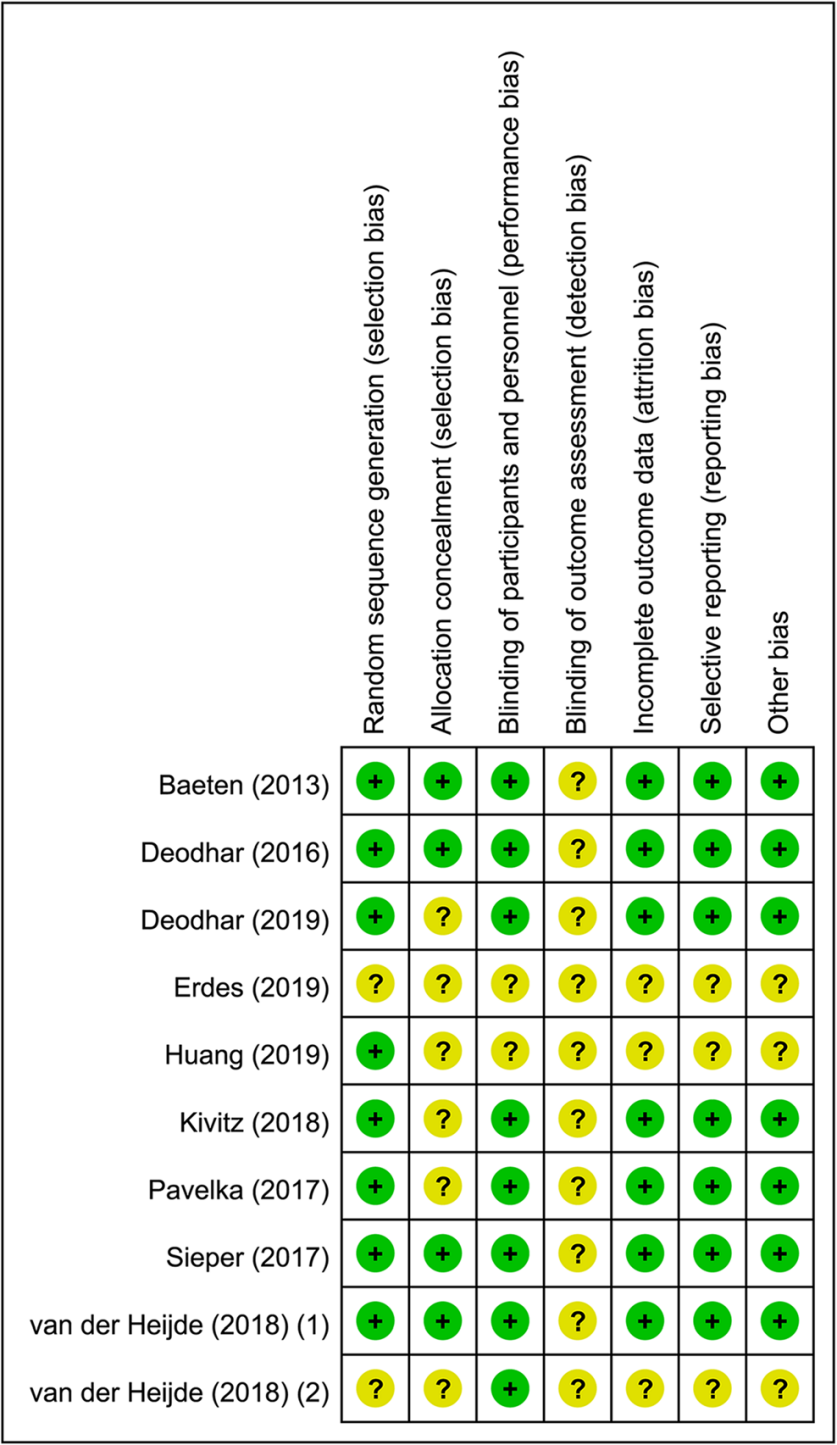
The quality of included studies evaluated by the Cochrane Collaboration’s tool

Among ten eligible studies in this meta-analysis, there were six studies for secukinumab, two studies for ixekizumab, one study for netakimab, and one study for bimekizumab. Characteristics of included studies were summarized in Table 1. In summary, (1) the year of publication ranged from 2013 to 2019; (2) the sample size ranged from 30 to 458; (3) the time to primary endpoint was 6 weeks for Baeten (2013) [32], 12 weeks for van der Heijde (2018) (2) [41], and 16 weeks for other eight studies; (4) mean age of patients ranged from 40.1 to 47.4 years; (5) the ratio of males ranged from 52.6 to 84.5%; (6) mean disease duration ranged from 5.2 to 13 years; (7) eight studies [33–35, 37–41] had different treatment arms based on dosage; (8) nine studies [32–34, 36–41] declared that they were sponsored. After the time of primary endpoint, all patients in both the treatment groups and the placebo groups entered an extended treatment period and received IL-17A inhibitors treatment. It should be noted that one patient in the treatment group of Baeten (2013) [32] was excluded from the efficacy analysis but included in the safety analysis due to a dosing error. The quality and the risks of bias of included studies were presented in Fig. 2. Notably, there were three RCTs being reported in conference abstracts respectively, and comprehensive information and data were not available.

**Table 1:** Characteristics of included studies in the meta-analysis

Author (year)	ClinicalTrials.gov identifier	Region	Time to primary endpoint (weeks)	Study duration (weeks)	Medication	Number of cases	Age (years)^a^	Males ratios (%)	Disease duration (years)^a^	HLA-B27 positive rate (%)	Baseline BASDAI^a^
Baeten (2013) [32]	NCT00809159	8 centers in Europe	6	28	SEC 2× 10 mg/kg	24	41.1 (10.1)	58	10.1 (12.2)	70	7.1 (1.40)
					placebo	6	45.0 (10.0)	83	10.2 (12.0)	83	7.2 (1.76)
Deodhar (2016) [33]	NCT01358175	106 centers	16	52	SEC 150 mg	125	40.1 (11.6)	67	6.5 (6.9)	69	6.4 (1.6)
					SEC 75 mg	124	42.3 (13.2)	71	7.9 (9.7)	80	6.1 (1.4)
					placebo	122	43.1 (12.4)	70	8.3 (8.9)	74	6.5 (1.5)
Deodhar (2019) [34]	NCT02696798	106 centers in 15 countries	16	52	IXE 80 mg Q2W	98	44.2 (10.8)	76.5	11.7 (8.8)	NA	7.5 (1.3)
					IXE 80 mg Q4W	114	47.4 (13.4)	79.8	10.1 (7.8)	NA	7.5 (1.3)
					placebo	104	46.6 (12.7)	83.7	13.0 (10.5)	NA	7.3 (1.3)
Erdes (2019) [35]	NA	NA	16	NA	NTK 40 mg	22	NA	NA	NA	NA	NA
					NTK 80 mg	22	NA	NA	NA	NA	NA
					NTK 120 mg	22	NA	NA	NA	NA	NA
					placebo	23	NA	NA	NA	NA	NA
Huang (2019) [36]	NCT02896127	China, Czech Republic, South Korea and the UK	16	52	SEC 150 mg	305	NA	NA	NA	NA	NA
					placebo	153	NA	NA	NA	NA	NA
Kivitz (2018) [37]	NCT02159053	85 centers in 19 countries	16	104	SEC 150 mg with load	116	44.5 (11.6)	69.8	8.4 (10.8)	86.2	7.0 (1.2)
					SEC 150 mg without load	117	41.2 (11.1)	70.9	6.5 (7.6)	84.6	6.95 (1.3)
					placebo	117	43.4 (12.5)	65	7.1 (9.2)	79.5	7.1 (1.2)
Pavelka (2017) [38]	NCT02008916	54 centers across the America and Europe	16	52	SEC 300 mg	76	42.1 (11.8)	65.8	5.3 (7.3)	73.7	7.0 (1.4)
					SEC 150 mg	74	42.9 (11.1)	62.2	6.0 (7.2)	70.3	7.0 (1.4)
					placebo	76	42.7 (11.4)	52.6	5.2 (6.4)	69.7	6.9 (1.3)
Sieper (2016) [39]	NCT01649375	106 centers	16	52	SEC 150 mg	72	41.9 (12.5)	64	7.0 (8.2)	79	6.6 (1.5)
					SEC 75 mg	73	44.4 (13.1)	70	5.3 (7.4)	73	6.6 (1.3)
					placebo	74	43.6 (13.2)	76	6.4 (8.9)	78	6.8 (1.3)
van der Heijde (2018) (1) [40]	NCT02696785	84 centers in 12 countries	16	52	IXE 80 mg Q2W	83	41.3 (11.2)	77	8.2 (9.0)	90	6.7 (1.6)
					IXE 80 mg Q4W	81	41.0 (12.1)	84	8.3 (9.6)	93	6.8 (1.3)
					placebo	87	42.7 (12.0)	83	6.8 (7.6)	89	6.8 (1.2)
van der Heijde (2018) (2) [41]	NCT02963506	NA	12	48	BIM 16 mg	243	42.2 (11.8)	84.5	NA	NA	NA
					BIM 64 mg				NA	NA	NA
					BIM 160 mg				NA	NA	NA
					BIM 320 mg				NA	NA	NA
					placebo	60			NA	NA	NA

*SEC*, secukinumab; *IXE*, ixekizumab; *NTK*, netakimab; *BIM*, bimekizumab; *BASDAI*, Bath AS Disease Activity Index; *Q2W*, every 2 weeks; *Q4W*, every 4 weeks

^a^Data were shown by mean and SD

### Efficacy of IL-17A inhibitors

#### ASAS20 response

**Fig. 3:**
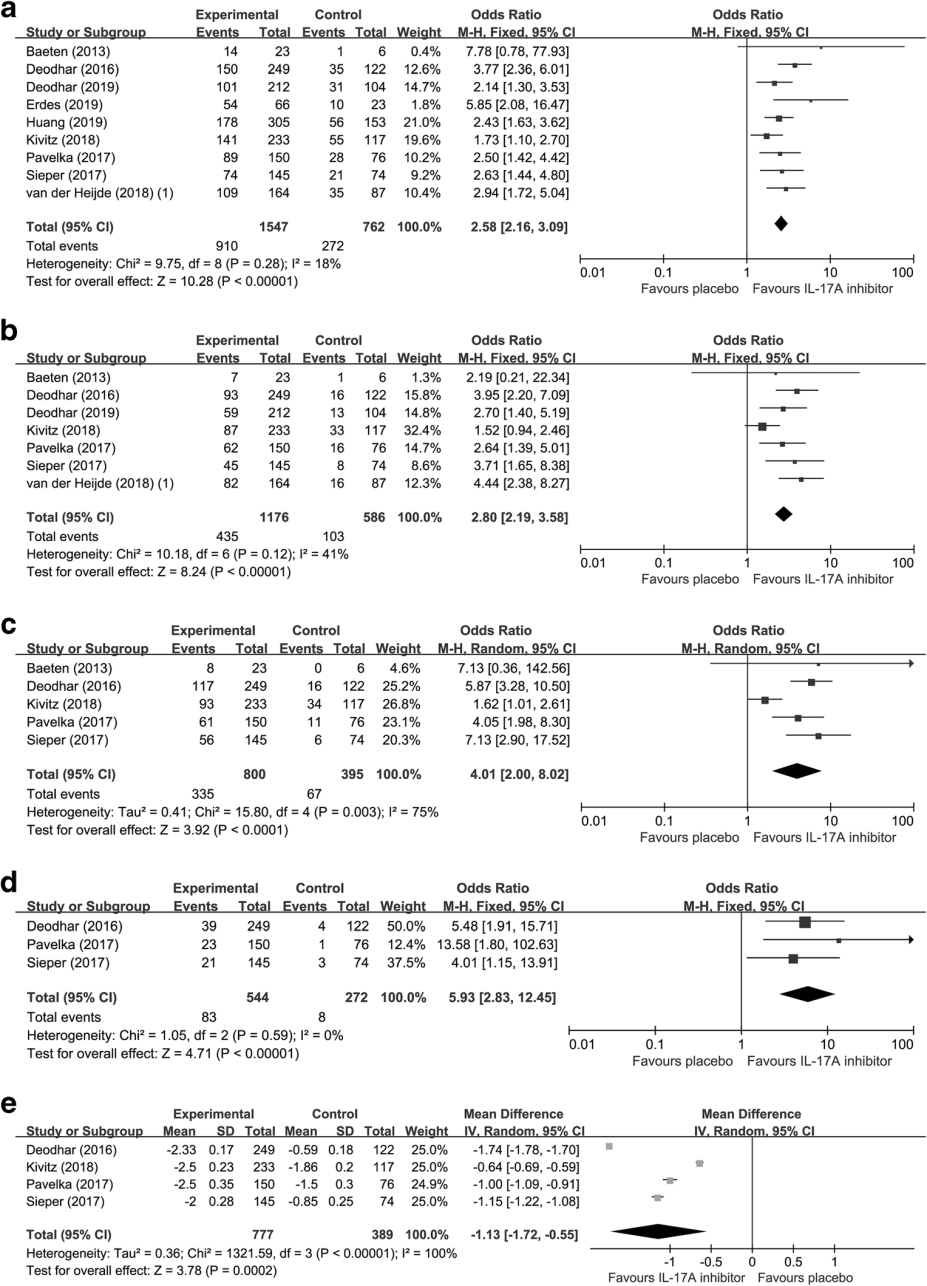
The efficacy evaluation of IL-17A inhibitors in AS. Forest plots of ORs for ASAS20 response (A), ASAS40 response (B), ASAS5/6 response (C), and ASAS partial remission (D) in patients with AS. Forest plots of MD for BASDAI (E) in patients with AS

ASAS20 response was defined as an outcome that patients achieved 20% improvement according to ASAS criteria. As a primary efficacy outcome indicator for AS, ASAS20 response was reported in nine studies consisting of 2309 patients. No significant heterogeneity among the RCTs was detected (*I*^2^ = 18%), and thus a fixed-effects model was performed. Compared with placebo, IL-17A inhibitors significantly improved the rate of ASAS20 response (OR = 2.58; 95% CI, 2.16 to 3.09; *p* < 0.01) (Fig. 3A). Subgroup analyses were performed according to three factors: medication (secukinumab, ixekizumab, or netakimab), time to primary endpoint (6 weeks or 16 weeks), and data source (full text or conference abstract) (Table 2). The results indicated that the efficacy of IL-17A inhibitors on ASAS20 response kept significant in the subgroup analyses. Furthermore, we performed sensitivity analysis and the result showed that the statistical significance was not altered after omitting any of the studies, further confirming the stability and credibility of the eventual results.

**Table 2:** The results of subgroup analyses by factors of medication, time to primary endpoint, and data source

Outcome	Subgroup	No. of studies	No. of patients	OR (95% CI)	*P* value
Medication					
ASAS20	SEC	6	1653	2.54 (2.05, 3.14)	< 0.01
	IXE	2	567	2.47 (1.72, 3.57)	< 0.01
	NET	1	89	5.85 (2.08, 16.47)	< 0.01
ASAS40	SEC	5	1195	2.54 (1.90, 3.41)	< 0.01
	IXE	2	567	3.49 (2.22, 5.48)	< 0.01
AEs	SEC	5	1196	1.20 (0.94, 1.53)	0.15
	IXE	2	567	1.44 (1.01, 2.05)	0.04
	BIM	1	303	0.93 (0.52, 1.66)	0.81
Nasopharyngitis	SEC	5	1196	1.84 (1.16, 2.94)	0.01
	IXE	2	567	1.39 (0.63, 3.06)	0.41
SAEs	SEC	6	1654	0.87 (0.47, 1.61)	0.66
	IXE	2	567	0.86 (0.30, 2.49)	0.78
Time to endpoint					
ASAS20	6 weeks	1	29	7.78 (0.78, 77.93)	0.08
	16 weeks	8	2280	2.56 (2.14, 3.07)	< 0.01
ASAS40	6 weeks	1	29	2.19 (0.21, 22.34)	0.51
	16 weeks	6	1733	2.81 (2.19, 3.59)	< 0.01
AEs	6 weeks	1	30	1.21 (0.04, 33.21)	0.91
	12 weeks	1	303	0.93 (0.52, 1.66)	0.81
	16 weeks	6	1733	1.27 (1.04, 1.56)	0.02
Nasopharyngitis	6 weeks	1	30	5.57 (0.28, 112.01)	0.26
	16 weeks	6	1733	1.66 (1.11, 2.50)	0.01
SAEs	6 weeks	1	30	0.83 (0.03, 22.87)	0.91
	16 weeks	7	2191	0.87 (0.51, 1.49)	0.61
Data source					
ASAS20	Full text	7	1762	2.55 (2.07, 3.13)	< 0.01
	Conference abstract	2	547	2.70 (1.86, 3.92)	< 0.01
AEs	Full text	7	1763	1.27 (1.04, 1.56)	0.02
	Conference abstract	1	303	0.93 (0.52, 1.66)	0.81
SAEs	Full text	7	1763	0.74 (0.41, 1.33)	0.32
	Conference abstract	1	458	1.69 (0.46, 6.25)	0.43

*SEC*, secukinumab; *IXE*, ixekizumab; *NTK*, netakimab; *BIM*, bimekizumab

#### ASAS40 response

ASAS40 response was also a crucial outcome indicator which represented patients achieved 40% improvement according to ASAS criteria. Seven studies with 1762 patients were included in the analysis of ASAS40 response. A fixed-effects model was more appropriate due to non-significant heterogeneity (*I*^2^ = 41%). IL-17A inhibitors made a significant improvement on ASAS40 response rate in AS (OR = 2.80; 95% CI, 2.19 to 3.58; *p* < 0.01) (Fig. 3B). We further conducted subgroup analyses and we found that the efficacy of IL-17A inhibitors on ASAS40 response remained significant and the outcomes were presented in Table 2. Additionally, sensitivity analysis was performed and the result remained significant after omitting single study one by one.

#### Other efficacy outcomes

There were five studies reporting data for ASAS5/6 response, four studies for the change from baseline in total BASDAI, and three studies for ASAS partial remission. Random effect model was suitable for the analyses of ASAS5/6 response (*I*^2^ = 75%) and the change from baseline in total BASDAI (*I*^2^ = 100%) owing to obvious heterogeneity. However, in the analysis of ASAS partial remission (*I*^2^ = 0%), no heterogeneity between the RCTs was detected and thus the fixed-effects model was performed. The results indicated that IL-17A inhibitors could significantly improve ASAS5/6 response rate (OR = 4.01; 95% CI, 2.00 to 8.02; *p* < 0.01) (Fig. 3C) and ASAS partial remission rate (OR = 5.93; 95% CI, 2.83 to 12.45; *p* < 0.01) (Fig. 3D), and reduce BASDAI score (MD = − 1.13; 95% CI, − 1.72 to − 0.55; *p* < 0.01) (Fig. 3E), indicating the favorable curative effects of IL-17A inhibitors in AS. Sensitivity analyses showed that removing any of the studies did not influence the results significantly.

### Safety of IL-17A inhibitors

#### AEs

**Fig. 4:**
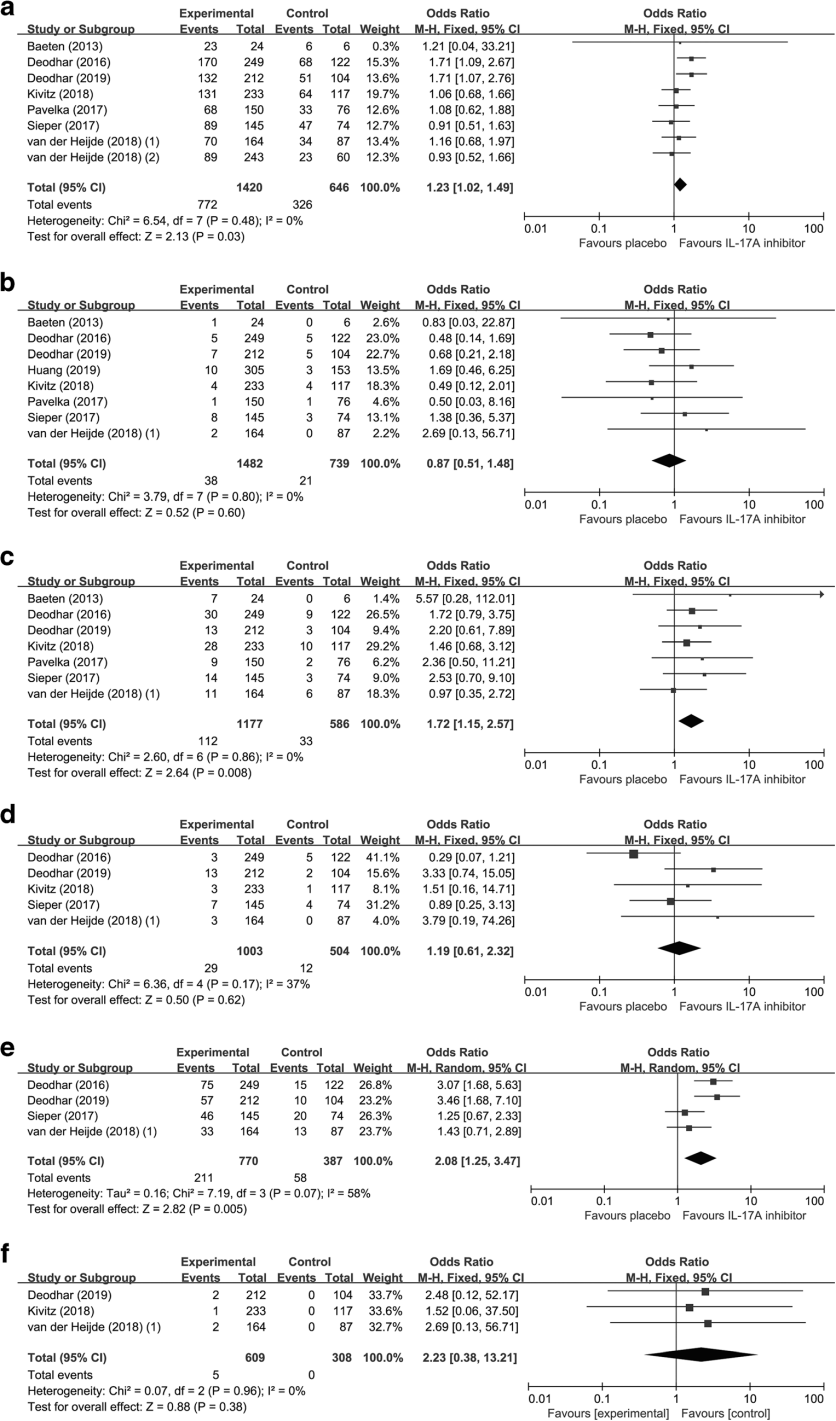
The safety evaluation of IL-17A inhibitors in AS. Forest plots of ORs for AEs (A), SAEs (B), nasopharyngitis (C), DDAAEs (D), infections (E), and serious infections

Eight studies with 2066 patients were included in the analysis of AEs. No heterogeneity between the RCTs was detected (*I*^2^ = 0%) and a fixed-effects model was performed. Comparing to placebo, IL-17A inhibitors increased risk of AEs (OR = 1.23; 95% CI, 1.02 to 1.49; *p* = 0.03) (Fig. 4A). We performed subgroup analyses and found that ixekizumab (OR = 1.44; 95% CI, 1.01 to 2.05; *p* = 0.04) rather than secukinumab (OR = 1.20; 95% CI, 0.94 to 1.53; *p* = 0.15) increased the risk of AEs (Table 2). When we removed Deodhar (2016) [33] or Deodhar (2019) [34], the results were altered to be insignificant (*p* = 0.21 or 0.18, respectively), indicating that increased risk of AEs might be derived from these two studies.

#### SAEs

Eight studies with 2221 patients were included in the analysis of SAEs. A fixed-effects model was performed in the analysis of SAEs (*I*^2^ = 0%). Meta-analysis of SAEs demonstrated that IL-17A inhibitors did not increase the risk of SAEs (OR = 0.87; 95% CI, 0.51 to 1.48; *p* = 0.60) (Fig. 4B). We further conducted subgroup analyses. The result did not indicate statistically significant differences between secukinumab and placebo (OR = 0.87; 95% CI, 0.47 to 1.61; *p* = 0.66), as well as ixekizumab and placebo (OR = 0.86; 95% CI, 0.30 to 2.49; *p* = 0.41) (Table 2). Removing any of the studies did not influence the result significantly.

#### Nasopharyngitis

Nasopharyngitis was the most commonly reported adverse event among these studies. Seven studies with 1763 patients were included in the analysis of nasopharyngitis. No heterogeneity among the RCTs was detected (*I*^2^ = 0%) and a fixed-effects model was performed. Comparing to placebo, IL-17A inhibitors increased the risk of nasopharyngitis (OR = 1.72; 95% CI, 1.15 to 2.57; *p* < 0.01) (Fig. 4C). The result of subgroup analyses indicated that secukinumab (OR = 1.84; 95% CI, 1.16 to 2.94; *p* = 0.01) rather than ixekizumab (OR = 1.39; 95% CI, 0.63 to 3.06; *p* = 0.41) increased the risk of nasopharyngitis (Table 2). Removing any of the studies did not influence the result significantly.

#### DDAAEs and deaths

There were five studies reporting data for DDAAEs. No heterogeneity was detected in the analysis of DDAAEs (*I*^2^ = 37%) and a fixed-effects model was performed. IL-17A inhibitors showed no impact on DDAAEs (OR = 1.19; 95% CI, 0.61 to 2.32; *p* = 0.62) (Fig. 4D). As for deaths, one death occurred in the placebo group in Deodhar (2016) [33] due to depression and suicide, one death occurred in the treatment group of secukinumab 75 mg in Sieper (2016) [39], and one death occurred in the treatment group of ixekizumab 80 mg every 2 weeks (Q2W) in Deodhar (2019) as a result of depression and suicide [34]. The impact of IL-17A inhibitors on the risk of death was not analyzed owing to limited data.

#### Infections and serious infections

Four studies were included for the analysis of infections. Significant heterogeneity was detected in the analysis of infections (*I*^2^ = 58%) and the random-effects model was performed. IL-17A inhibitors increased the risk of infections (OR = 2.08; 95% CI, 1.25 to 3.47; *p* < 0.01) (Fig. 4E). In addition, there were totally 5 serious infections among the included patients: 1 patient in the treatment group of secukinumab 150 mg with a loading regimen (secukinumab 150 mg with load) in Kivitz (2018) [37], 2 patients in treatment group of ixekizumab 80 mg every 4 weeks (Q4W) in Deodhar (2019) [34], and 2 patient in the treatment groups in van der Heijde (2018) (1) [40]. Meta-analysis of serious infections did not indicate statistically significant differences between treatment and control groups (OR = 2.23; 95% CI, 0.38 to 13.21; *p* = 0.38) (Fig. 4F).

## Discussion

AS, a part of a larger class of spondyloarthropathies, is a relatively common, chronic, and serious autoimmune disease mainly affecting the axial skeleton and spinal joints, eventually reducing the quality of life, also extorting heavy economic burdens and pressure on individuals and the society. As the disease progresses, ankylosis is a major characteristic of AS in advanced cases, leading to the fusion of vertebrae, decreased mobility, and increased long-term disability. Notably, although there are many available treatments to effectively relieve the symptoms of AS, there is no cure method currently. The treatment options for patients with AS were really limited. In recent years, the potential application of bDMARDs has gradually attracted extensive attention of researchers, and the clinical management of AS has been significantly changed as a result of the introduction of bDMARDs.

IL-17, mainly produced by Th17 cells, plays a crucial role in protecting hosts from bacterial and fungal infections under physiological condition. In addition, IL-17 could improve the expression of various proinflammatory cytokines and further lead to inflammatory activation. Recently, IL-17A inhibitors were proved to be novel bDMARDs for several autoimmune diseases, including psoriasis [28], rheumatoid arthritis [29, 30], and inflammatory bowel disease [31]. Overexpressed IL-17 was also highly involved in the autoimmune dysfunction and the disease progression in AS patients. However, the clinical value of IL-17A inhibitors is still controversial. To our best knowledge, the current meta-analysis is the first to comprehensively evaluate the efficacy and safety of IL-17A inhibitors in patients with AS.

Through pooling the data of 10 independent RCTs and comprehensively analyzing the ASAS20 response, ASAS40 response, ASAS5/6 response, and ASAS partial remission, we found that IL-17A inhibitors significantly alleviated the clinical signs and symptoms of AS. In addition, disease activity was controlled by IL-17A inhibitors as measured by decreased BASDAI. Significant heterogeneity was detected in the analysis of ASAS5/6 response and BASDAI, which may reduce the credibility of the data to some extent. Furthermore, after the time of primary endpoint, all patients in both the treatment groups and the placebo groups entered an extended treatment period and received IL-17A inhibitors. There are 7 extension studies reporting that secukinumab possesses sustained efficacy in AS through 1 to 5 years [45–51]. Similarly, the extension studies of ixekizumab demonstrated that the efficacy through 52 weeks was consistent with 16 weeks [52]. Therefore, IL-17A inhibitors demonstrated favorable curative effects in patients with AS and were expected to be promising drugs for AS.

AEs, nasopharyngitis, and infections were found to be more frequently in treatment groups compared with placebo. However, there was no evidence that IL-17A inhibitors increased the risk of SAEs, serious infections, and DDAAEs. As for the death risk, OR was not obtained owing to limited data. To be specific, there were totally three deaths in the included trials, two of which happened in the treatment groups. Furthermore, IL-17A inhibitors may increase the risk of neutropenia owing to the immunosuppressive properties of these biologics [33, 34]. Therefore, attention should be paid to this problem when applying IL-17A inhibitors in the treatment of AS. Since this finding was based on a relatively small number of studies, the results should be interpreted prudently. Likewise, the safety of IL-17A inhibitors was controversial in other autoimmune diseases. A meta-analysis of twenty-seven RCTs showed that IL-17A inhibitors increased the risk of AEs in moderate-to-severe plaque psoriasis [53]. In addition, the risk of AEs but not SAEs was increased by IL-17A inhibitors in patients with psoriasis and psoriatic arthritis [54]. However, Kunwar et al. performed a meta-analysis of seven RCTs about rheumatoid arthritis and reported that IL-17A inhibitors did not significantly increase the risk of AEs or SAEs in rheumatoid arthritis [55]. Therefore, the safety of IL-17A inhibitors remains to be further studied.

As a consequence of the introduction of bDMARDs, TNF inhibitors were considered as usual first-line bDMARDs treatment. However, due to intolerance and inefficacy of TNF inhibitors, IL-17A inhibitors as a novel bDMARDs were recommended for AS patients who do not respond to TNF inhibitors according to *2016 update of the ASAS-EULAR management recommendations for axial spondyloarthritis* [10]. Moreover, a cost-effectiveness analysis in Finland indicated that secukinumab was less costly and more effective compared to adalimumab, certolizumab pegol, etanercept, golimumab, and infliximab in AS treatment [56]. Also, secukinumab was proved to be the most cost-effective treatment option versus other biologics in AS by numerous researches conducted in Canada [57], Argentina [58], the UK [59], and Russia [60].

Besides the 10 included RCTs, more trials are planned or conducted to further evaluate the efficacy and safety of IL-17A inhibitors in AS. In detail, there are three ongoing or planned studies for secukinumab (identifier: NCT03259074, NCT02763046, NCT03350815), two for bimekizumab (identifier: NCT03215277 and NCT03355573), and one for netakimab (identifier: NCT03447704). Because these studies are not completed, we have had no access to the relevant data so far. Therefore, we will pay close attention to the progress of these studies in the future.

Some potential limitations in our study should be acknowledged. First, we could not obtain the comprehensive information and data of three included RCTs reported in three conference abstracts respectively [35, 36, 41]. Second, the number of RCTs included in this study was still relatively small, especially for a certain type of medication, there were only two studies available for ixekizumab, one study for netakimab and one study for bimekizumab. More trials are warranted in the future to further assess the efficacy and safety of IL-17A inhibitors in AS. Third, dose-effect relationship of IL-17A inhibitors was not analyzed due to various kinds of medications and limited trials for each. In addition, different treatment arms in eight included studies were combined into one treatment group respectively.

In conclusion, the results from this meta-analysis showed that IL-17A inhibitors significantly improved clinical signs and symptoms of AS, which supported the application of IL-17A inhibitors in the treatment of AS. However, studies with larger sample size and longer follow-up times are firmly warranted to evaluate the safety of IL-17A inhibitors in AS.

## Data Availability

Not applicable.

## Abbreviations

IL: interleukin
AS: ankylosing spondylitis
RCT: randomized controlled trial
ASAS: the Assessment of SpondyloArthritis International Society
ASAS20: 20% improvement according to ASAS criteria
ASAS40: 40% improvement according to ASAS criteria
BASDAI: Bath AS disease activity index
OR: odds ratio
MD: mean difference
AE: adverse event
SAE: serious adverse event
CI: confidence interval
DDAAEs: discontinuation due to any AEs
HLA: human leukocyte antigen
NSAID: non-steroidal anti-inflammatory drug
bDMARD: biological disease-modifying antirheumatic drugs
TNF: tumor necrosis factor
CTLA8: cytotoxic T lymphocyte antigen 8
IL-17R: IL-17 receptor
NF-κB: nuclear factor κB
MAPK: mitogen-activated protein kinase
C/EBP: CCAAT/enhancer-binding protein
Th: T helper
SEC: secukinumab
IXE: ixekizumab
NTK: netakimab; BIM: bimekizumab

## References

[1] Sieper J, Poddubnyy D (2017). Axial spondyloarthritis. Lancet.

[2] Braun J, Sieper J (2007). Ankylosing spondylitis. Lancet.

[3] Dougados M, Baeten D (2011). Spondyloarthritis. Lancet.

[4] Stolwijk C, van Onna M, Boonen A, van Tubergen A (2016). Global prevalence of spondyloarthritis: a systematic review and meta-regression analysis. Arthritis Care Res.

[5] Sag S, Nas K, Sag MS (2018). Relationship of work disability between the disease activity, depression and quality of life in patients with ankylosing spondylitis. J Back Musculoskelet Rehabil.

[6] Kruger K, von Hinuber U, Meier F (2018). Ankylosing spondylitis causes high burden to patients and the healthcare system: results from a German claims database analysis. Rheumatol Int.

[7] de BJ, Polman A, De B-M (1961). Hereditary factors in rheumatoid arthritis and ankylosing spondylitis. Ann Rheum Dis.

[8] Brewerton DA, Hart FD, Nicholls A, Caffrey M, James DC, Sturrock RD (1973). Ankylosing spondylitis and HL-A 27. Lancet.

[9] Dagfinrud, H., T.K. Kvien, and K.B. Hagen (2008) Physiotherapy interventions for ankylosing spondylitis. Cochrane Database Syst Rev Cd002822 10.1002/14651858.CD002822.pub3 PMC8453259 18254008

[10] Van Der Heijde, D., S. R Ramiro (2017) Annals of the Rheumatic Diseases 76:978–991 10.1136/annrheumdis-2016-210770 28087505

[11] Ward MM, Deodhar A, Akl EA, Lui A, Ermann J, Gensler LS, Smith JA, Borenstein D, Hiratzka J, Weiss PF, Inman RD, Majithia V, Haroon N, Maksymowych WP, Joyce J, Clark BM, Colbert RA, Figgie MP, Hallegua DS, Prete PE, Rosenbaum JT, Stebulis JA, van den Bosch F, Yu DTY, Miller AS, Reveille JD, Caplan L (2016). American College of Rheumatology/Spondylitis Association of America/Spondyloarthritis Research and Treatment Network 2015 recommendations for the treatment of ankylosing spondylitis and nonradiographic axial spondyloarthritis. Arthritis Rheumatol.

[12] Bunte, K. and T. Beikler (2019) Th17 Cells and the IL-23/IL-17 axis in the pathogenesis of periodontitis and immune-mediated inflammatory diseases. Int J Mol Sci 20 10.3390/ijms20143394 PMC6679067 31295952

[13] Lubberts E (2015). The IL-23-IL-17 axis in inflammatory arthritis. Nat Rev Rheumatol.

[14] Suzuki E, Mellins ED, Gershwin ME, Nestle FO, Adamopoulos IE (2014). The IL-23/IL-17 axis in psoriatic arthritis. Autoimmun Rev.

[15] Rouvier E, Luciani MF, Mattei MG (1993). CTLA-8, cloned from an activated T cell, bearing AU-rich messenger RNA instability sequences, and homologous to a herpesvirus saimiri gene. J Immunol.

[16] Taams LS, Steel KJA, Srenathan U, Burns LA, Kirkham BW (2018). IL-17 in the immunopathogenesis of spondyloarthritis. Nat Rev Rheumatol.

[17] Gaffen SL (2009). Structure and signalling in the IL-17 receptor family. Nat Rev Immunol.

[18] Frleta M, Siebert S, McInnes IB (2014). The interleukin-17 pathway in psoriasis and psoriatic arthritis: disease pathogenesis and possibilities of treatment. Curr Rheumatol Rep.

[19] Zhu S, Qian Y (2012). IL-17/IL-17 receptor system in autoimmune disease: mechanisms and therapeutic potential. Clin Sci (Lond).

[20] Shen H, Goodall JC, Hill Gaston JS (2009). Frequency and phenotype of peripheral blood Th17 cells in ankylosing spondylitis and rheumatoid arthritis. Arthritis Rheum.

[21] Wendling D, Cedoz JP, Racadot E, Dumoulin G (2007). Serum IL-17, BMP-7, and bone turnover markers in patients with ankylosing spondylitis. Joint Bone Spine.

[22] Jandus C, Bioley G, Rivals JP, Dudler J, Speiser D, Romero P (2008). Increased numbers of circulating polyfunctional Th17 memory cells in patients with seronegative spondylarthritides. Arthritis Rheum.

[23] Limon-Camacho L, Vargas-Rojas MI, Vazquez-Mellado J (2012). In vivo peripheral blood proinflammatory T cells in patients with ankylosing spondylitis. J Rheumatol.

[24] Kenna TJ, Davidson SI, Duan R, Bradbury LA, McFarlane J, Smith M, Weedon H, Street S, Thomas R, Thomas GP, Brown MA (2012). Enrichment of circulating interleukin-17-secreting interleukin-23 receptor-positive gamma/delta T cells in patients with active ankylosing spondylitis. Arthritis Rheum.

[25] Mei Y, Pan F, Gao J, Ge R, Duan Z, Zeng Z, Liao F, Xia G, Wang S, Xu S, Xu J, Zhang L, Ye D (2011). Increased serum IL-17 and IL-23 in the patient with ankylosing spondylitis. Clin Rheumatol.

[26] Appel H, Maier R, Wu P, Scheer R, Hempfing A, Kayser R, Thiel A, Radbruch A, Loddenkemper C, Sieper J (2011). Analysis of IL-17(+) cells in facet joints of patients with spondyloarthritis suggests that the innate immune pathway might be of greater relevance than the Th17-mediated adaptive immune response. Arthritis Res Ther.

[27] Dubash S, Bridgewood C, McGonagle D, Marzo-Ortega H (2019). The advent of IL-17A blockade in ankylosing spondylitis: secukinumab, ixekizumab and beyond. Expert Rev Clin Immunol.

[28] Leonardi C, Matheson R, Zachariae C, Cameron G, Li L, Edson-Heredia E, Braun D, Banerjee S (2012). Anti-interleukin-17 monoclonal antibody ixekizumab in chronic plaque psoriasis. N Engl J Med.

[29] Genovese MC, Durez P, Richards HB, Supronik J, Dokoupilova E, Mazurov V, Aelion JA, Lee SH, Codding CE, Kellner H, Ikawa T, Hugot S, Mpofu S (2013). Efficacy and safety of secukinumab in patients with rheumatoid arthritis: a phase II, dose-finding, double-blind, randomised, placebo controlled study. Ann Rheum Dis.

[30] Hueber, W., D.D. Patel, T. Dryja, et al (2010) Effects of AIN457, a fully human antibody to interleukin-17A, on psoriasis, rheumatoid arthritis, and uveitis. Sci Transl Med 2:52ra72 10.1126/scitranslmed.3001107 20926833

[31] Targan SR, Feagan B, Vermeire S, Panaccione R, Melmed GY, Landers C, Li D, Russell C, Newmark R, Zhang N, Chon Y, Hsu YH, Lin SL, Klekotka P (2016). A randomized, double-blind, placebo-controlled phase 2 study of brodalumab in patients with moderate-to-severe Crohn’s disease. Am J Gastroenterol.

[32] Baeten D, Baraliakos X, Braun J, Sieper J, Emery P, van der Heijde D, McInnes I, van Laar JM, Landewé R, Wordsworth P, Wollenhaupt J, Kellner H, Paramarta J, Wei J, Brachat A, Bek S, Laurent D, Li Y, Wang YA, Bertolino AP, Gsteiger S, Wright AM, Hueber W (2013). Anti-interleukin-17A monoclonal antibody secukinumab in treatment of ankylosing spondylitis: a randomised, double-blind, placebo-controlled trial. Lancet.

[33] Deodhar AA, Dougados M, Baeten DL, Cheng-Chung Wei J, Geusens P, Readie A, Richards HB, Martin R, Porter B (2016). Effect of secukinumab on patient-reported outcomes in patients with active ankylosing spondylitis a phase III randomized trial (MEASURE 1). Arthritis & Rheumatology.

[34] Deodhar A, Poddubnyy D, Pacheco-Tena C, Salvarani C, Lespessailles E, Rahman P, Järvinen P, Sanchez-Burson J, Gaffney K, Lee EB, Krishnan E, Santisteban S, Li X, Zhao F, Carlier H, Reveille JD, COAST-W Study Group (2019). Efficacy and safety of ixekizumab in the treatment of radiographic axial spondyloarthritis: sixteen-week results from a phase III randomized, double-blind, placebo-controlled trial in patients with prior inadequate response to or intolerance of tumor necrosis factor inhibitors. Arthritis and Rheumatology.

[35] Erdes S, Nasonov E, Kunder E et al (2019) Primary efficacy of netakimab, a novel interleukin-17 inhibitor, in the treatment of active ankylosing spondylitis in adults. Clin Exp Rheumatol 31025924

[36] Huang, F., F. Sun, W. Wan, et al (2019) Secukinumab provides rapid and significant improvement in the signs and symptoms of ankylosing spondylitis: primary (16-week) results from a phase 3 China-centric study, measure 5. Annals of the Rheumatic Diseases 78 (Supplement 2):894-895

[37] Kivitz AJ, Wagner U, Dokoupilova E, Supronik J, Martin R, Talloczy Z, Richards HB, Porter B (2018). Efficacy and safety of secukinumab 150 mg with and without loading regimen in ankylosing spondylitis: 104-week results from MEASURE 4 study. Rheumatology and Therapy.

[38] Pavelka, K., A. Kivitz, E. Dokoupilova, et al (2017) Efficacy, safety, and tolerability of secukinumab in patients with active ankylosing spondylitis: a randomized, double-blind phase 3 study, MEASURE 3. Arthritis Research and Therapy 19 (1) (no pagination) 10.1186/s13075-017-1490-y PMC5741872 29273067

[39] Sieper J, Deodhar A, Marzo-Ortega H, Aelion JA, Blanco R, Jui-Cheng T, Andersson M, Porter B, Richards HB, MEASURE 2 Study Group (2017). Secukinumab efficacy in anti-TNF-naive and anti-TNF-experienced subjects with active ankylosing spondylitis: results from the MEASURE 2 study. Ann Rheum Dis.

[40] van der Heijde D, Cheng-Chung Wei J, Dougados M, Mease P, Deodhar A, Maksymowych WP, van den Bosch F, Sieper J, Tomita T, Landewé R, Zhao F, Krishnan E, Adams DH, Pangallo B, Carlier H, COAST-V study group (2018). Ixekizumab, an interleukin-17A antagonist in the treatment of ankylosing spondylitis or radiographic axial spondyloarthritis in patients previously untreated with biological disease-modifying anti-rheumatic drugs (COAST-V): 16 week results of a phase 3 randomised, double-blind, active-controlled and placebo-controlled trial. Lancet.

[41] Van Der Heijde, D., L.S. Gensler, A. Deodhar, et al (2019) Dual neutralisation of IL-17A and IL-17F with bimekizumab was associated with improvements in patient-reported and quality-of-life outcomes in patients with active ankylosing spondylitis: results from a phase 2B, randomised, double-blind, placebo-controlled, dose-ranging study. Annals of the Rheumatic Diseases 78 (Supplement 2):193

[42] Ungprasert P, Erwin PJ, Koster MJ (2017). Indirect comparisons of the efficacy of biological agents in patients with active ankylosing spondylitis: a systematic review and meta-analysis. Clin Rheumatol.

[43] Chen C, Zhang X, Xiao L, Zhang XS, Ma XL (2016). Comparative effectiveness of biologic therapy regimens for ankylosing spondylitis: a systematic review and a network meta-analysis. Medicine (Baltimore).

[44] Cumpston, M., T. Li, M.J. Page, et al (2019) Updated guidance for trusted systematic reviews: a new edition of the Cochrane Handbook for Systematic Reviews of Interventions. Cochrane Database Syst Rev 10:Ed000142 10.1002/14651858.ED000142 PMC10284251 31643080

[45] Baraliakos X, Kivitz AJ, Deodhar AA, Braun J, Wei JC, Delicha EM, Talloczy Z, Porter B, MEASURE 1 Study Group (2018). Long-term effects of interleukin-17A inhibition with secukinumab in active ankylosing spondylitis: 3-year efficacy and safety results from an extension of the phase 3 MEASURE 1 trial. Clin Exp Rheumatol.

[46] Braun J, Baraliakos X, Deodhar A, Poddubnyy D, Emery P, Delicha EM, Talloczy Z, Porter B (2019). Secukinumab shows sustained efficacy and low structural progression in ankylosing spondylitis: 4-year results from the MEASURE 1 study. Rheumatology (Oxford).

[47] Baraliakos X, Braun J, Deodhar A, Poddubnyy D, Kivitz A, Tahir H, van den Bosch F, Delicha EM, Talloczy Z, Fierlinger A (2019). Long-term efficacy and safety of secukinumab 150 mg in ankylosing spondylitis: 5-year results from the phase III MEASURE 1 extension study. RMD Open.

[48] Braun J, Baraliakos X, Deodhar A, Baeten D, Sieper J, Emery P, Readie A, Martin R, Mpofu S, Richards HB, MEASURE 1 study group (2017). Effect of secukinumab on clinical and radiographic outcomes in ankylosing spondylitis: 2-year results from the randomised phase III MEASURE 1 study. Ann Rheum Dis.

[49] Baraliakos X, Borah B, Braun J, Baeten D, Laurent D, Sieper J, Emery P, McInnes IB, van Laar JM, Wordsworth P, Wollenhaupt J, Kellner H, Colin L, Vandenhende F, Radford K, Hueber W (2016). Long-term effects of secukinumab on MRI findings in relation to clinical efficacy in subjects with active ankylosing spondylitis: an observational study. Ann Rheum Dis.

[50] Marzo-Ortega H, Sieper J, Kivitz A, Blanco R, Cohen M, Martin R, Readie A, Richards HB, Porter B, on behalf of the Measure 2 Study Group (2017). Secukinumab and sustained improvement in signs and symptoms of patients with active ankylosing spondylitis through two years: results from a phase III study. Arthritis Care Res (Hoboken).

[51] Marzo-Ortega H, Sieper J, Kivitz A, Blanco R, Cohen M, Delicha EM, Rohrer S, Richards H (2017). Secukinumab provides sustained improvements in the signs and symptoms of active ankylosing spondylitis with high retention rate: 3-year results from the phase III trial, MEASURE 2. RMD Open.

[52] Dougados, M., J.C. Wei, R. Landewe, et al (2019) Efficacy and safety of ixekizumab through 52 weeks in two phase 3, randomised, controlled clinical trials in patients with active radiographic axial spondyloarthritis (COAST-V and COAST-W). Ann Rheum Dis 10.1136/annrheumdis-2019-216118 PMC7025731 31685553

[53] Erichsen, C.Y., P. Jensen, and K. Kofoed (2019) Biologic therapies targeting the interleukin (IL)-23/IL-17 immune axis for the treatment of moderate-to-severe plaque psoriasis: a systematic review and meta-analysis. J Eur Acad Dermatol Venereol 10.1111/jdv.15879 31419343

[54] Loft, N.D., S. Vaengebjerg, A.S. Halling, et al (2019) Adverse events with IL-17 and IL-23 inhibitors for psoriasis and psoriatic arthritis: a systematic review and meta-analysis of phase III studies. J Eur Acad Dermatol Venereol 10.1111/jdv.16073 31721310

[55] Kunwar S, Dahal K, Sharma S (2016). Anti-IL-17 therapy in treatment of rheumatoid arthritis: a systematic literature review and meta-analysis of randomized controlled trials. Rheumatol Int.

[56] Purmonen T, Puolakka K, Mishra D, Gunda P, Martikainen J (2019). Cost-effectiveness of secukinumab compared to other biologics in the treatment of ankylosing spondylitis in Finland. Clinicoecon Outcomes Res.

[57] Goeree R, Chiva-Razavi S, Gunda P, Jain M, Jugl SM (2019). Cost-effectiveness analysis of secukinumab in ankylosing spondylitis from the Canadian perspective. J Med Econ.

[58] Citera, G., P.M. Bianculli, A. Khare, et al (2018) Cost-effectiveness of secukinumab versus other biologics in the treatment ofankylosing spondylitis: an Argentinean perspective. Journal of Clinical Rheumatology 24 (3 Supplement 1):S117

[59] Marzo-Ortega, H., A. Halliday, S. Jugl, et al (2017) The cost-effectiveness of secukinumab versus tumour necrosis factor a inhibitor biosimilars for ankylosing spondylitis in the UK. Rheumatology (United Kingdom) 56 (Supplement 2):ii92

[60] Fedyaev D, Derkach EV (2016). Cost-effectiveness analysis of different biologic agents for ankylosing spondylitis treatment in Russia. Value Health.

